# New Method Improves the Evaluation of Subclinical Left Ventricular Dysfunction in Type 2 Diabetes Mellitus

**DOI:** 10.5935/abc.20190164

**Published:** 2019-08

**Authors:** Lutfu Askin, Okan Tanrıverdi, Hakan Tibilli, Serdar Turkmen

**Affiliations:** Adiyaman Universitesi Egitim ve Arastirma Hastanesi - Cardiology, Adıyaman, Centry - Turkey

**Keywords:** Antioxidants/pharmacology, Apoptosis/drug effects, Diabetes Mellitus, Reactive Oxygen Species, Myocardial, Systole, Dyastole, Heart Failure

Tei et al.^1^ firstly described the myocardial performance index (MPI), which is showing both systolic and diastolic functions of the left ventricle. As a prognostic marker increased MPI has been shown to be an independent predictor of mortality and morbidity in various diseases such as myocardial infarction, hypertension, diabetes, and heart failure.^2,3^ Askin et al.^4^ showed that left ventricular (LV) diastolic and systolic functions were negatively affected in prediabetic patients. In addition, MPI can also be used in the assessment of abnormal cardiac function parameters in prediabetic patients. Furthermore, the most prominent feature of our method is that it can be obtained in a short period of time with easily available equipment. It is important to identify subclinical left ventricular diastolic dysfunction (LVDD) for clinical prevention before significant LVDD occurs. For this purpose, MPI is used to identify subclinical LVD in type 2 diabetes mellitus (DM).

Presystolic wave (PSW) measurement is obtained via doppler examination of LV outflow tract (LVOT).^5,6^ Kul et al.^7^ found that the PSW is associated with subclinical LVDD in patients with type 2 diabetes. PSW is an easily measurable echocardiographic parameter obtained in late diastole and can predict subclinical left ventricular dysfunction in patients with type 2 diabetes. Possible causes of PSW formation are impaired LV compliance and increased LV stiffness, which are also leading causes of PSW in diabetic patients among others. Furthermore, the relationship between PSW and LVDD has been proven.^5^

Stahrenberg et al.^8^ reported that LV diastolic dysfunction is associated with glucose metabolism in a broad spectrum from impaired glucose tolerance to overt diabetes. Simone et al.^9^ have recently reported that the risk of heart failure was increased markedly with type 2 diabetes, which was independent of myocardial infarction and hypertension (HT). Therefore; in the medical literature, the term ''diabetic cardiomyopathy'' has been proposed to be used in cases of ventricular dysfunction in the absence of coronary artery disease and HT.^10^

Hyperglycemia may result in the build-up of myocardial proteins via excessive accumulation of increased glycosylated products (AGE) and this may cause rigid myocardium. Accumulation of AGEs results in reduced myocardial relaxation by disrupting cross-links between collagen molecules. Hyperglycemia may also cause myocyte apoptosis, accelerated myocardial cell loss, decreased ventricular contraction, and systolic dysfunction. In conclusion, these phenomena cause decreased LV systolic and diastolic functions in diabetic patients.^11,12^
